# 

**Published:** 1896-02

**Authors:** 


### CONTENTS FOR FEBRUARY, 1896.

ORIGINAL COMMUNICATIONS. PAGE.

Treatment of Compound Fractures. By EUGENE A. SMITH, M. D. .......... 529
Appendicitis. By S. W. S. TOMS, M. D. .......... 537
Irrigation of the Peritoneal Cavity. By JOHN T. PITKIN, M. D. .......... 548
Historical Sketch of the Medical Society of the County of Erie—1821-1896. By FRANKLIN C. GRAM, M. D. .......... 551
Localised Peritonitis. By C O. BAKER, M. D. .......... 559

CLINICAL REPORT.

A Few Interesting Cases .......... 561

TRANSLATION.

Dilatation of the Esophagus Caused by a Stricture in the Cardia .......... 564

SOCIETY PROCEEDINGS.

Medical Society of the County of Erie .......... 570
Medical Society of the County of Chautauqua .......... 574

PROGRESS IN MEDICAL SCIENCE.

Surgery .......... 575
State Medical Examinations .......... 582

EDITORIAL.

Medical Society of the County of Erie .......... 587
Topics of the Month .......... 589

---

Personal .......... 595
Obituary .......... 596
Society Meetings .......... 597
Hospital Notes .......... 598
Reviews .......... 599
Literary Notes .......... 605
Miscellany .......... 608

